# Simulating Population Genetics of Pathogen Vectors in Changing Landscapes: Guidelines and Application with *Triatoma brasiliensis*

**DOI:** 10.1371/journal.pntd.0003068

**Published:** 2014-08-07

**Authors:** Francois Rebaudo, Jane Costa, Carlos E. Almeida, Jean-Francois Silvain, Myriam Harry, Olivier Dangles

**Affiliations:** 1 BEI-UR072, IRD, Gif-sur-Yvette, France; 2 LEGS-UPR9034, CNRS-UPSud11, Gif-sur-Yvette, France; 3 Laboratório de Biodiversidade Entomológica, Instituto Oswaldo Cruz - Fiocruz, Rio de Janeiro, Rio de Janeiro, Brasil; 4 Departamento de Ciências Biológicas, Faculdade de Ciências Farmacêuticas, UNESP, Araraquara, Sao Paolo, Brasil; 5 Instituto de Ecología, Campus Cotacota, Universidad Mayor San Andrés, La Paz, Bolivia; IRD/CIRDES, Burkina Faso

## Abstract

**Background:**

Understanding the mechanisms that influence the population dynamics and spatial genetic structure of the vectors of pathogens infecting humans is a central issue in tropical epidemiology. In view of the rapid changes in the features of landscape pathogen vectors live in, this issue requires new methods that consider both natural and human systems and their interactions. In this context, individual-based model (IBM) simulations represent powerful yet poorly developed approaches to explore the response of pathogen vectors in heterogeneous social-ecological systems, especially when field experiments cannot be performed.

**Methodology/Principal Findings:**

We first present guidelines for the use of a spatially explicit IBM, to simulate population genetics of pathogen vectors in changing landscapes. We then applied our model with *Triatoma brasiliensis*, originally restricted to sylvatic habitats and now found in peridomestic and domestic habitats, posing as the most important *Trypanosoma cruzi* vector in Northeastern Brazil. We focused on the effects of vector migration rate, maximum dispersal distance and attraction by domestic habitat on *T. brasiliensis* population dynamics and spatial genetic structure. Optimized for *T. brasiliensis* using field data pairwise fixation index (*FST*) from microsatellite loci, our simulations confirmed the importance of these three variables to understand vector genetic structure at the landscape level. We then ran prospective scenarios accounting for land-use change (deforestation and urbanization), which revealed that human-induced land-use change favored higher genetic diversity among sampling points.

**Conclusions/Significance:**

Our work shows that mechanistic models may be useful tools to link observed patterns with processes involved in the population genetics of tropical pathogen vectors in heterogeneous social-ecological landscapes. Our hope is that our study may provide a testable and applicable modeling framework to a broad community of epidemiologists for formulating scenarios of landscape change consequences on vector dynamics, with potential implications for their surveillance and control.

## Author Summary

Worldwide, humans are modifying landscapes at an unprecedented rate. These modifications have an influence on the ecology of pathogen vectors, yet this issue has received relatively little input from modeling research. The current study presents guidelines for the use of a modeling framework for the representation of the dynamics and spatial genetic structure of pathogen vectors. It allows considering spatiotemporal landscape modifications explicitly, to represent human-altered modifications and consequences. We applied this modeling framework to *Triatoma brasiliensis*, vector of the pathogen *Trypanosoma cruzi* responsible for the Chagas disease, in the semi-arid Northeastern Brazil. Using field data of pairwise fixation index (*FST*) from microsatellite loci, we found that migration rate, maximum dispersal distance and attraction by domestic habitat were all key parameters to understand vector spatial genetic structure at the landscape level. At the interface across disciplines, this study provides to the community of epidemiologists a testable and applicable framework to foresee landscape modification consequences on vector dynamics and genetic structure, with potential implications for their surveillance and control.

## Introduction

Human-induced landscape changes are increasingly recognized as important drivers of infectious disease outbreak and emergence events, resulting in significant threats to public health [1]–[3]. Worldwide the rapid modification of natural habitats has triggered intense research on the landscape epidemiology of vector diseases to describe how the temporal dynamics of host, vector, and pathogen populations interact spatially within heterogeneous and changing environments to enable transmission (see [4] for a review). Landscape changes not only affect the transmission of endemic infections by modifying contact patterns between hosts and vectors [5], but also have an effect on selection pressure, leading to the dominance of pathogen strains and vector populations adapted to new environmental conditions [4]. While evolutionary ecologists have increasingly recognized the importance of evolutionary processes (e.g., local adaptive genetic variation in pathogen vectors) to predict population response to changing landscape conditions [6], this issue has received relatively little attention among landscape epidemiologists. As a result, we are lacking spatially and temporally explicit quantitative approaches required to understand the key causal mechanisms (*e.g.*, habitat selection and adaptation, migration and resulting gene flow) involved in pathogen vector response to landscape changes [7]. This is particularly true for pathogen vectors in tropical regions where landscape changes (deforestation, urbanization) occur at an accelerated rate [8] putting at risk human populations with limited resources to face disease-related challenges.

The main objective of this study is to propose a methodological framework to simulate spatial population genetics of pathogen vectors in heterogeneous and changing landscapes, in order to link observed patterns with processes. We adapted the SimAdapt simulation software [9] to simulate the evolution of both neutral and adaptive genotypes of diploid, sexually reproducing pathogen vectors introduced into a landscape. This genetic model accounts for vector dispersal and adaptation to local conditions and is coupled to a cellular automaton allowing the representation of land-use and land-cover changes. It includes landscape features known to influence vectors' genetic structure (e.g., roads, domestic, peridomestic and sylvatic habitats linked to possible loci under selection). Model simulations can be compared to field data of vector's spatial genetic structure. As a step further, the model can be used to simulate the evolution of vector spatial genetic structure in changing landscapes (e.g., prospective scenario of deforestation or urbanization).

After providing general guidelines on our model, we applied SimAdapt to explore local adaptation processes of pathogen vectors in a real-world landscape using *Triatoma brasiliensis* Neiva, 1911 (Hemiptera, Reduviidae, Triatominae) as a study model. *T. brasiliensis* is a blood sucking bug vector of the pathogen responsible for the Chagas disease (American trypanosomiasis), caused by a parasite *Trypanosoma cruzi* (Kinetoplastea, Trypanosomatidae). This disease affects approximately 10 million people in Latin America and Caribbean [10], and is recognized by the World Health Organization as one of the world's most neglected tropical disease [11]. *T. brasiliensis* represents the most important vector of *T. cruzi* in Northeastern Brazil [12], [13]. As for several species of native Triatominae (*e.g.*, *Rhodnius equatorialis,* [14]; *T. pseudomaculata*, [15], [16]), *T. brasiliensis* was originally restricted to sylvatic habitats but since its description it has been increasingly found invading and establishing in peridomestic and domestic habitats [17]–[20]. Among all Brazilian triatomines, *T. brasiliensis* is the one that exhibits the highest pressure for re-infestation after insecticide house spraying. As a consequence, six months (sometimes longer) after chemical treatment, human dwellings start being re-infested [21], [22]. As a native vector, its eradication would require significant logistical and technical investment in the long term, and, therefore, control efforts are kept on insecticide house spraying and improvement.

Here, we used SimAdapt to explore three parameters related to the dispersal of *T. brasiliensis*: i) the migration rate; ii) the dispersal distance (*i.e.* the maximum distance covered by an individual during its life cycle, including passive and active dispersal); and iii) the attraction by domestic habitat (*i.e.* the strength that avoid individual from emigrating, as a consequence of attraction by light or availability of hosts to feed on). Model simulations were then compared to field data of *T. brasiliensis* population genetic structure and prospective scenarios of landscape change effects on *T. brasiliensis* adaptation were run.

## Materials and Methods

### The SimAdapt model

#### Digital representation of the landscape

**Figure 1:**
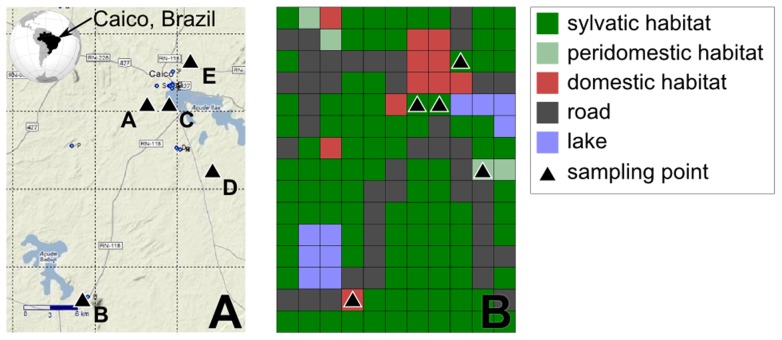
Study landscape (A) and matrix representation of the landscape in simAdapt (B). The different habitat types, roads and lakes are represented by different colors. The black triangles represent the location of sample points from the field data in the Caicó municipality in Northeastern Brazil.

The first step to perform simulations in SimAdapt is to build a digital representation of the study landscape. To achieve this goal, the landscape is categorized into habitat types. Optionally, as SimAdapt selection submodel can be set to consider that being adapted to a given habitat is considered advantageous while the individual is in this habitat, but disadvantageous in others habitats (see [23] for a discussion on alternative selection models), the categorization of habitat type can reflect a selective pressure resulting in a differential reproductive fitness of individuals. Once categorized into habitat types, the landscape is converted into a matrix representation. In this matrix, habitat types are converted into a discrete variable made of grid cells (raster representation, see Figure 1 in the study case below) unlike in a geographic information system (GIS), where delimited areas (here habitat types) are typically drawn continuously (vectorial representation). The tessellation defines the resolution of the landscape which should be defined according to the ecological characteristics of the vector model, in particular its dispersal capabilities. At this point, each cell of the grid corresponding to a habitat type can be parameterized with a carrying capacity and a resistance to vectors' emigration. The carrying capacity defines the maximum number of individuals that can be located into a cell, and the resistance for migration defines the permeability of the landscape (*sensu* [24]) ranging from 0 (*i.e.*, no resistance) to 100 (*i.e.*, impermeable barrier) − see [1], [25] for reviews on the effects of landscape resistance on infectious diseases and vectors. Technically, any GIS software provides the necessary features to define areas (habitat types), as well as additional layers for carrying capacity and resistance for migration. This information can be exported into SimAdapt as three different “ascii” files at a chosen resolution. Alternatively, the map can be built entirely from the graphical user interface (see Appendix S1 in [9]).

#### Initialization of the simulation model

The second step is to initialize the parameters of the model as presented in Table 1. Depending on available knowledge on vector species, unknown parameters can be estimated within a range of possible values (referred as “parameter space”), and sensitivity analyses performed to evaluate the influence of parameter variation on simulations output results.

**Table 1:** Initialization of state variables in the SimAdapt model.

Description	Value	Justification/References
**Population dynamics submodel**		
Growth rate of the logistic model	0.3	[13]
* Migration rate (*m*)	[0.1:1.0]	[18]
* Maximum dispersal distance (*d*)	[1:5]	[29]
* Attraction by domestic habitat (*l*)	[1:10]	[15], [51]
**Genetic submodel**		
Number of microsatellites loci	7	[21]
Heterozygosity at initialization (*H_0_*)	0.5±0.2	To fit the field sample
Mutation rate for microsatellites	10e-4	Default value [46]
Number of loci under selection per habitat type	1	Default value [9]
Coefficients of selection (*s* and *h*)	s = 0.2h = 0.5	Assumption for codominance and strong selection (prospective scenarios only)
**Landscape representation**		
Carrying capacity matrix for the logistic growth model (adults)	50	Based on field sample [30], [31]
Resistance matrix for emigration	Heterogeneous	To fit the landscape
Habitat type matrix for natural selection	[1:3]	To fit the landscape
Location and number of individuals at initialization	everywhere(50 adult individuals)	To fit the field sample
Scenario of landscape change	Deforestation and urbanization	[8], [44], [45]
**Sampling submodel**		
Virtual sampling	Population sampled without replacement	According to field sampling design
Number of individuals sampled per sampling point	25	According to field sampling design
Number of points sampled	5	According to field sampling design
**Simulation**		
Number of repetitions per simulation	30	To account for stochasticity
Number of generations per simulation	100	Time to stabilize *FST*
Output files format	ARLEQUIN (Arlecore)	To fit the field sample analyzes

The three variables studied in this paper are highlighted with an asterisk at the beginning of the first column.

#### Optimization

The third step is to optimize model parameterization using field data. It consists in the comparison of studied parameters in their range of possible values (parameter space), with observed data. Parameter optimization can take several forms depending on available data. It can vary from standard genetic algorithms [26], or random search [27], to the use of simulations previously performed as sensitivity analysis.

#### Prospective scenarios

Once the model has been optimized, SimAdapt can perform prospective simulations to represent the effects of landscape changes (based on scenarios of land-use changes) on local adaptation of vector species. Technically, a user-defined scenario of land-use change is established in SimAdapt using the landscape cellular automaton submodel (see [9] for more details).

### The case of *Triatoma brasiliensis* in Northeastern Brazil

As an example of application of our model in the context of vector-borne tropical diseases, we used genetic field data on *T. brasiliensis* (vector of the pathogen responsible for the Chagas disease) in the Caicó municipality in Northeastern Brazil (Rio do Grande do Norte state). Triatomine individuals were sampled during a field work performed in March 2011. Domestic habitats consisted in houses, peridomestic habitats of the area 50 meters around houses and sylvatic habitats of areas at a minimum of 200 meters from any house. In total 126 individuals were collected in five different locations (populations) chosen to cover a range of distance between sampling points and habitat types (three sylvatic, one peridomestic and one domestic populations; see Figure 1A). All individuals were genotyped according to seven microsatellites as neutral markers [21], and pairwise fixation index (*FST*), allelic diversity and observed heterozygosity computed using Arlequin version 3.5 [28]. A detailed description is available in supporting information S1, and *FST* results in Table 2.

**Table 2:** Pairwise fixation index (*FST*) between sampling points A, B, C, D and E (in bold italic when the associated *p-value* was below 0.05), for the observed dataset in the Caicó municipality in Northeastern Brazil.

	A	B	C	D	E
A	0				
B	***0.02130***	0			
C	**0.01513**	***0.02265***	0		
D	0.00635	0.00852	***0.02791***	0	
E	0.00000	***0.03253***	***0.02440***	***0.01609***	0

As the main objective of our study case was to explore the effect of vector dispersal on observed population genetic structure (using *FST* values), we focused our analysis on three specific variables: i) vector's migration rate (*m*); ii) vector's dispersal distance (*i.e.*, the maximum distance covered by an individual during its life cycle, including passive and active dispersal (*d*)); and iii) vector's attraction by a specific habitat type (*i.e.*, the strength that impedes individual from emigrating when they are located in a specific habitat type (*l*)). Note that all output files from simulations were designed as input files for Arlequin (genotypes of individuals from the 7 microsatellite loci), using the same sampling method, so that observed and simulated *FST* values could be compared.

#### Study landscape and its digital representation

The study landscape, in the semi-arid Northeastern Brazil, part of the municipality of Caicó, was represented as a matrix of 11 per 15 grid cells (see Figure 1). In this tessellation, one grid cell is equivalent to 2*2 km. Grid cells were categorized according to *T. brasiliensis* ecology [12], [19] to include domestic habitats, peridomestic habitats, and sylvatic habitats (see [18] for a description and discussion). Physical barriers to dispersal and establishment (lakes) and potential factors of passive dispersal by human (roads), were also considered and georeferenced. This allowed us to apply a matrix of landscape resistance to our grid. Based on previous studies on *T. infestans* by [29], the maximal active dispersal distance was set to 2 km per generation over the entire landscape. To simulated passive dispersal, the dispersal distance on roads was fixed to a maximum of 10 km per generation (corresponding to *d* = 5, with *d* varying from 1 to 5 grid cells in our simulations).

#### Initialization of the simulation model

We set up our landscape with 50 adult individuals per grid cell, within the range of Triatominae densities documented in the literature ([30], [31]). Note that using more individuals per grid cell reduced the variance in simulation results, due to a reduction in genetic drift, but did not change observed patterns. Individuals were attributed genotypes using 7 microsatellite loci with alleles randomly chosen in a normal distribution. We did not account for selection as analyses performed from *F*-statistics on field data suggested no loci under selection (using Arlequin based on [32]). The other parameters were set to their default value in SimAdapt or according to available literature (see Table 1). The three variables related to dispersal (*m*, *d*, and *l*) were defined as ranges of potential values (parameter space; see Table 1) and then further explored through a sensitivity analysis (see below).

#### Model validation

**Figure 2:**
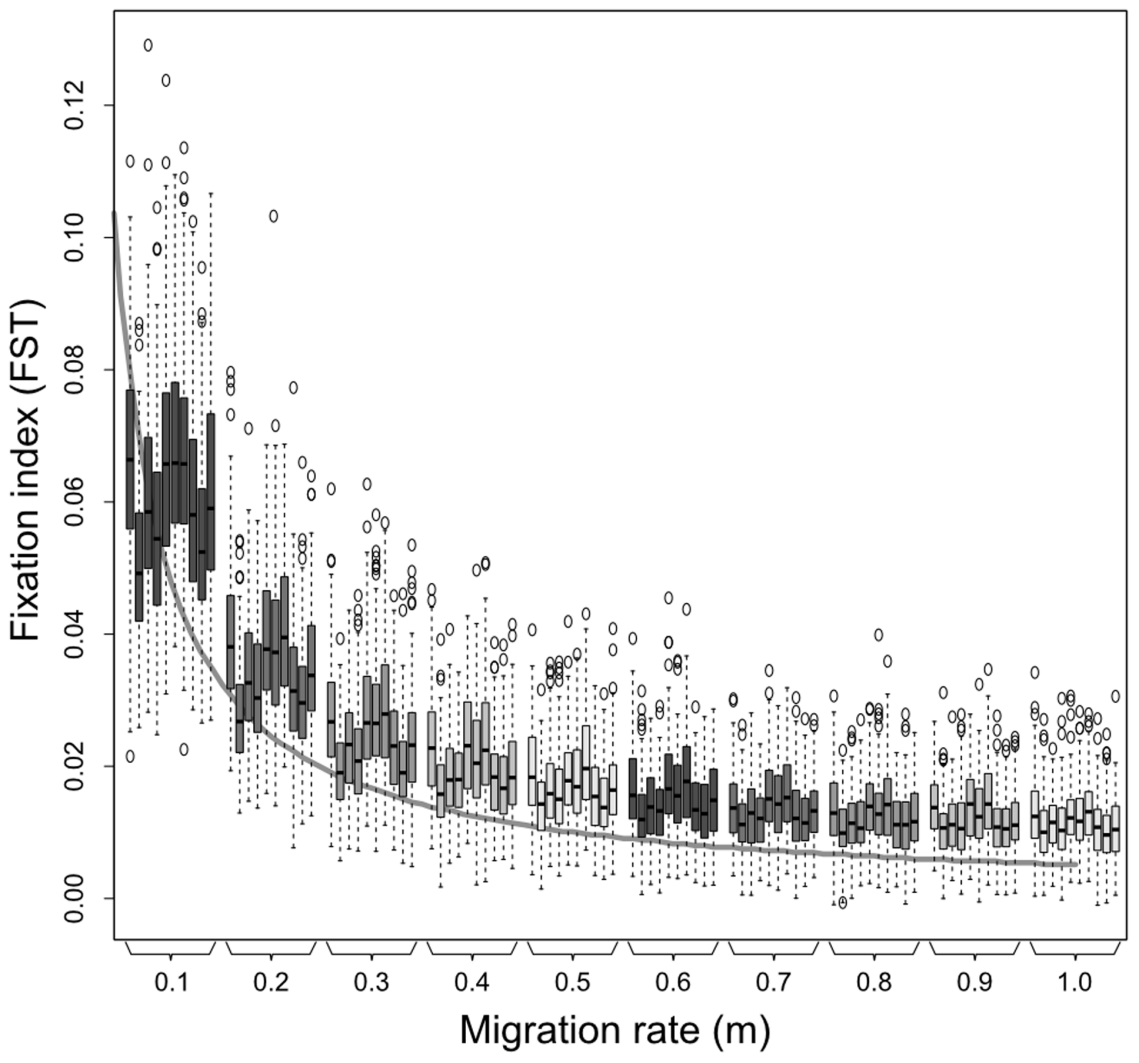
Validation of the model initialization using the response of *FST* to migration rate. *FSTs* between sampling points (from left to right for each migration rate: AB, AC, AD, AE, BC, BD, BE, CD, CE, DE), are represented as a function of migration rate from 0.1 to 1. Simulated results are represented using boxplots of 30 repetitions, for all values of dispersal distance (*d* ranging from 1 to 5 by 1), *i.e.* 150 pairwise *FST* values per boxplot. The theoretical expectation is represented by a solid grey line (FST ≈ 1/(4*Nm*+1) with *N* = 50 and *m* ranging continuously from 0 to 1).

Validation is an important process in model development, and consist in the demonstration that the model meets performance standards under specific conditions [33], [34]. To validate SimAdapt initialization for *T. brasiliensis*, we calculated *FST* using Arlequin (based on genotypes from the 7 microsatellite loci and assuming no selection for habitat type) and compared its values with *FST* from theoretical expectation in an island model [35]. In an island model, the theoretical response of *FST* to migrant number fits a curve of the form *FST* ≈ 1/(4*Nm*+1), where *N* is the effective population size and *m* is the migration rate between populations. Our case slightly differed from the island model as it included a finite number of populations and dispersal mechanisms partially driven by the habitat types. We consequently expected different *FST* values than those predicted by theory [36], [37]. We found a significant relationship between FST and *Nm* using a nonlinear least squares model for migration rate from 0.1 to 1 (FST  =  1/(5.85*Nm*+1.24), *p<0.05*, see Figure 2). This result confirmed that, within the range of parameters' space, our simulation model behaved according to theoretical expectations thereby validating its use for *T. brasiliensis*.

#### Sensitivity analyses

Sensitivity analyses were performed to explore the effect of three variables related to vector dispersal on triatomine genetic differentiation coefficient (*FST*): i) the effect of migration rate from *m* = 0.1 to 1 per generation; ii) the effect of maximum dispersal distance from *d* = 1 to 5 grid cells per generation; and iii) the effect of attraction by domestic habitat from *l* = 0 to 10 (*l* representing a factor that reduce emigration from domestic habitat, *i.e.* when *l* = 10, *m* = *m*/10). The resulting sets of simulations were repeated 30 times to account for stochasticity. We performed an additional set of 30 repetitions to ensure that the number of repetitions was representative of stochastic processes (Student tests, *T_mean_*  = 0.39, *df_mean_*  = 296.65, *p_min_>0.23*). All analyses were performed on a random sampling of 25 individuals at the five sampling locations (*i.e.*, 125 individuals in total; see [38] for a discussion on virtual sampling). Genotypes of sampled individuals for the 7 microsatellite loci were analyzed to calculate *FST* after 100 generations using Arlecore from Arlequin version 3.5 [28]. This time was sufficient to observe a stabilization of the *FST* for all sets of simulations. Analyses of variance (ANOVA) were run to evaluate the effect of migration rate (*m*), dispersal distance (*d*) and domestic habitat attraction (*l*) on the *FST* using R software [39].

#### Model optimization

Model outputs from the sensitivity analysis were compared to those obtained from field data using least-square optimization [40] between simulated and observed *FST*. The optimization procedure generated 300 *FST* values (10 migration rates values x 5 dispersal distances values x 6 domestic habitat attraction values), repeated 30 times. Based on this data, we determined the values of migration rate, maximum dispersal distance and attraction by domestic habitat that minimized the difference between simulated and observed *FST* data. Additionally, we plotted a ternary plot using an Akima interpolation [41] on the mean values of the 30 repetitions to visualize the topography of the least-square optimization. To evaluate the goodness of fit of our simulation, we compared simulated and observed *FST* values using one-sample Student tests (see [42], [43] for examples of *FST* comparisons). The values that minimized difference between simulated and observed data were then used to initialize the prospective scenario.

#### Prospective scenarios

**Figure 3:**
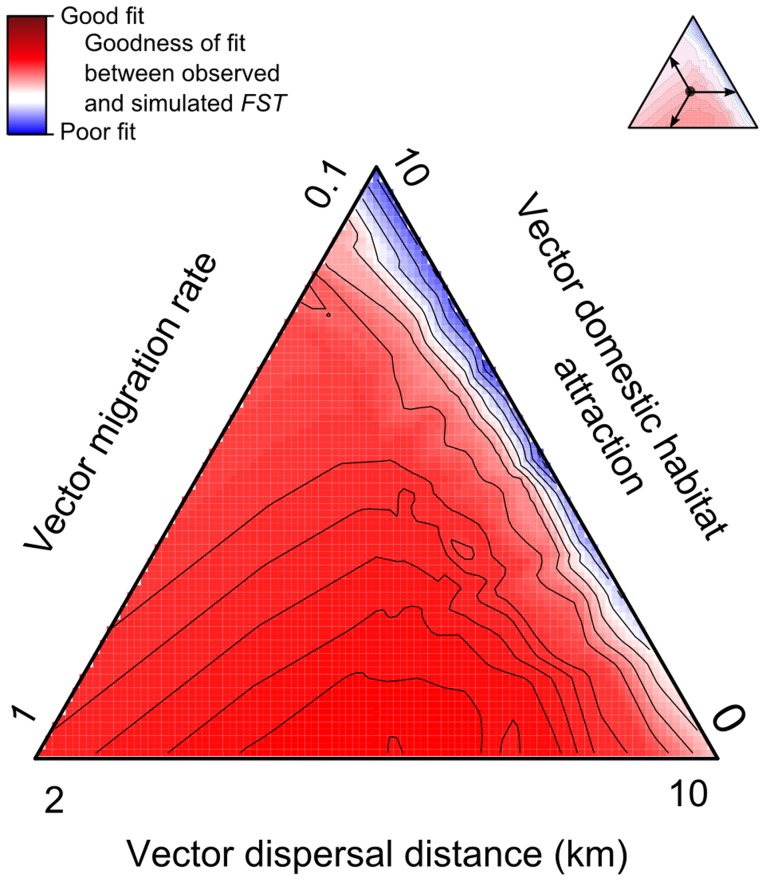
Goodness of fit between observed and simulated *FST* for *T. brasiliensis* in Northeastern Brazil. The red from blue color gradient represents an Akima interpolation of the least-square optimization between observed and simulated *FST* for different values of vector migration rate, dispersal distance and domestic habitat attraction. *FST* were computed using Arlequin over the 10 couples of sampled points. Sets of simulations were repeated 30 times for each value of migration rate (*m* index ranging from 0.1 to 1 by 0.1), dispersal distance (*d* ranging from 2 to 10 km by 2 km) and domestic habitat attraction (*l* index ranging from 0 to 10 by 2). The plot is represented using mean values with a gradient from blue (high value, *i.e.*, poor fit), to red (low value, *i.e.*, good fit).

We performed forward-time simulations of the potential effects on *T. brasiliensis* population genetics of landscape change (urbanization) within the next 50 years. Our simplified scenario of urbanization consisted in the progression of peridomestic and domestic habitats over sylvatic habitats (1 km per year to mimic worst case scenario of deforestation, see [44], [45] for a discussion).The prospective scenarios included selection considering one locus under selection per habitat type (domestic, peridomestic and sylvatic), with two alleles (“generalist” and “specialized”, see [9]). As selection coefficient and degree of dominance (*s* and *h*, see [46]) were unknown and because our field sampling did not allow to infer such parameters (no time series of alleles frequencies, see [47]), the degree of dominance was represented to reproduce co-dominance (*h* = 0.5), and the selection coefficient to reproduce strong selection (*s* = 0.2) (see [46]). *T. brasiliensis* dispersal variables were set up using results from the optimization procedure (*m* = 0.6; *d* = 3; *l* = 2; see Figure 3). The simulations were run over 100 triatomine generations, with one generation equivalent to approximately 6 months ([13]). Individuals were sampled every generation at all sampling points, and *FST* computed over time using Arlecore (see Table 1 for parameterization). Four scenarios were compared using two factors (land-use change and selection) and two states (with/without) using two-way analysis of variance of *FST* after 100 generations.

## Results

### Sensitivity analyses

As expected, we found a significant effect of migration rate (*m*) on the *FST* values (see Table 3). The effect of dispersal distance (*d*) was also significant except between two sampling sites (A and C) that were very close from each other (Euclidian distance; *F_AC_*_(1, 8991)_ = 1.4, *p = 0.24*). Dispersal distance explained much less variance in the ANOVA than migration rate (see differences in *F-values* in Table 3). The same result was found when testing for the effect of domestic habitat attraction on *FST*. On average, for all 10 couples of sampled locations, 58%±7 of the variance of *FST* in the ANOVA models was explained by migration rate, dispersal distance, and domestic type attraction.

**Table 3:** Analysis of variance (ANOVA) of the effect of migration rate, dispersal distance, and domestic habitat attraction on *FST*.

	Df	Sum Sq	Mean Sq	F value	p-value
migration rate (m)	1	22.725	22.725	67252	<0.05
dispersal distance (d)	1	0.097	0.097	288	<0.05
domestic habitat attraction (l)	1	2.960	2.960	8759	<0.05
interaction m:d	1	0.012	0.012	35	<0.05
interaction m:l	1	0.053	0.053	157	<0.05
Residuals	89982	30.406	0.0003		

*FST* were computed between the 10 couples of locations for the 300 parameters combinations, with 30 repetitions per combination.

### Optimization

The optimization procedure revealed that the difference between observed and simulated *FST* was lowest at high migration rates, average dispersal distances and low domestic habitat attractions (see the redder zone of the ternary plot, Figure 3). The best set of parameter values from this zone explained 50% of the *FST* values observed in the field (see Table 4, using *m* = 0.6; *d* = 3; and *l* = 2). Note that overall, the combinations of migration rate, dispersal distance and domestic habitat attraction could significantly explain 70% of *FST* values.

**Table 4:** Comparison of *FST* values between simulated and observed data using One-sample Student tests.

Couples of sampled locations	*m*	*d*	*l*	*p_mean_* (% >0.05)	T_mean_	*df*
AB*	[0.1:1]	[1:5]	[0:10]	0.113 (29%)	5.67	29
	0.6	3	2	0.867	−0.17	29
AC*	[0.1:1]	[1:5]	[0:10]	0.077 (20%)	4.50	29
	0.6	3	2	0.06	−1.96	29
AD	[0.1:1]	[1:5]	[0:10]	<0.01 (0%)	14.18	29
	0.6	3	2	<0.01	13.03	29
AE	[0.1:1]	[1:5]	[0:10]	<0.01 (0%)	25.14	29
	0.6	3	2	<0.01	23.06	29
BC*	[0.1:1]	[1:5]	[0:10]	0.097 (28%)	5.38	29
	0.6	3	2	0.325	−1.00	29
BD	[0.1:1]	[1:5]	[0:10]	<0.01 (0%)	14.98	29
	0.6	3	2	<0.01	12.66	29
BE*	[0.1:1]	[1:5]	[0:10]	0.030 (9%)	8.12	29
	0.6	3	2	0.624	0.49	29
CD	[0.1:1]	[1:5]	[0:10]	0.038 (10%)	−4.26	29
	0.6	3	2	<0.01	−12.03	29
CE*	[0.1:1]	[1:5]	[0:10]	0.020 (5%)	9.00	29
	0.6	3	2	0.864	0.17	29
DE	[0.1:1]	[1:5]	[0:10]	0.008 (3%)	16.37	29
	0.6	3	2	<0.01	10.08	29

For each couple of sampled locations, the first line represents average values for all combinations of parameters (*m*, *d* and *l*) with the percentage of significant combinations between brackets, and the second line for *m* = 0.6, *d* = 3 and *l* = 2. Couples of sampled locations significantly explained by this parameterization are shown in bold with an asterisk.

### Prospective scenarios of land-use change

**Figure 4:**
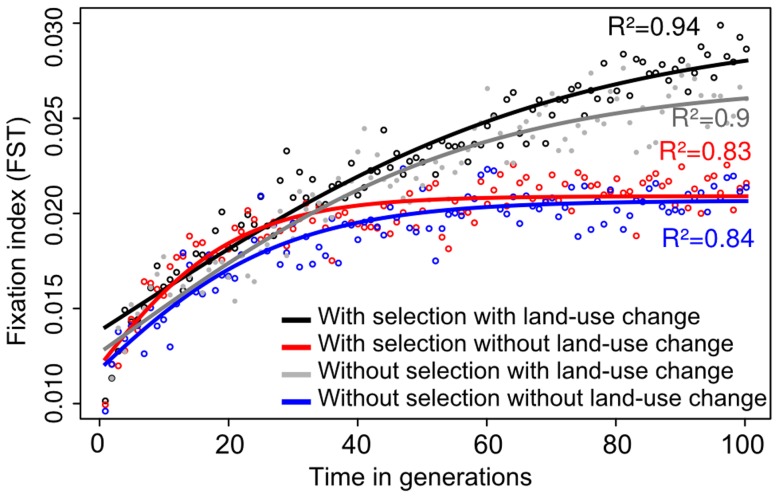
Evolution of *FST* through time between two sampled locations. Each point represents the mean of 30 repetitions. Curves were fitted to the general form of a sigmoid function using *nls* function in R. The different colors corresponds to scenarios with selection and with land-use change (in black); with selection and without land-use change (in red); without selection and without land-use change (in blue); and without selection and with land-use change (in grey).

The dynamics of the spatial genetic structure of *T. brasiliensis* populations strongly differed between the urbanization and the "no land-use change" (control) scenarios (see Figure 4 for *FST* between individuals located at sampling points A and B). In the control scenarios, *FST* reached a threshold and stabilized within 100 generations for all couples of sampled locations (*e.g.*, mean *FST* threshold value of 0.021 between A and B with and without selection, see Figure 4). Contrastingly, *FST* values did not reach such threshold in the urbanization scenarios (*e.g.*, mean *FST* threshold value of 0.030 and 0.027 between A and B with and without selection, respectively), except between individuals at sampling locations A, C and E. We found a significant effect of land-use change on *FST* after 100 generations (*e.g.*, between A and B: *F* = 24.6, *df* = 1, *p<0.05*). Contrastingly, the effect of selection was not significant, even if *FST* values were generally higher in the scenarios including selection.

## Discussion

Understanding the dispersal behavior of vector arthropods is a central issue in the control and surveillance of vector-borne tropical diseases [48], [49]. Although dispersal studies on vectors have been conducted since the eighties (*e.g.*, [29]), accurate descriptions of the spatio-temporal distribution of most tropical pathogen vectors are still lacking. Along with recent advances in spatially explicit models [48], our study proposes a contribution to better characterize the dispersal behavior of the vectors of *T. cruzi*, responsible for Chagas disease. This characterization is exemplified in this study with a generic platform providing a natural description of the dispersal mechanisms [50], adapted to the social-ecological system inhabited by *T. brasiliensis*. Our study successfully ranked parameters of *T. brasiliensis* dispersal and participated in explaining observed patterns (spatial population differentiation using *FST*) by linking them to processes (vectors ecology and behavior). It thus represents a contribution in understanding the underlying mechanisms of *T. brasiliensis* spatial genetic structure and population dynamics.

Inevitably, our study made a series of assumptions and simplifications inherent to the modeling process. If some were attributable to the methodological framework, others pointed gaps in the knowledge of *T. brasiliensis* ecology and genetics. The selection for habitat type submodel, for instance, relied on the theoretical basis of one locus under selection per habitat type. Further studies of *T. brasiliensis* are needed and should allow the identification candidate genes responsible for adaptation for habitat type which would considerably refine the theoretical submodel, and possibly lead to inference on selection coefficients. Moreover, additional field studies, such as those conducted for other Triatomine species (*e.g.*, studies describing and quantifying the influence on vectors of public street lights; [51], [52]; or describing the vectors active and passive dispersal [53]) would help refining our results for *T. brasiliensis*. Additionally, the use of a more complex population dynamic model might have permitted to analyze the impact of other demographic parameters on population structuring, like the density of the population or the lifespan. Consequently, prospective scenarios, grounded on actual knowledge and limited dataset, should be regarded as theoretical insights. They remain pertinent tools with implications in term of vectors surveillance and control. For example, our *T. brasiliensis* prospective scenarios in Northeastern Brazil revealed a significant influence of land-use on vector spatial genetic structure. It suggested that in urbanized areas, where hosts are abundant, vector population gene flow would be reduced (higher *FST* between sampling points). While anthropogenic landscape disturbance proved to increase vector infection by *T. cruzi* [54], it is more important than ever to anticipate for the effect of future landscapes on vector dynamics and spatial genetic structure, in order to establish efficient management strategies. This study on *T. brasiliensis* and associated prospective scenarios suggest that control techniques should be examined in their social-ecological context, accounting for anthropogenic features to come.

Beyond our empirical study case, the objective of the paper was to present a methodological framework for studying vectors population genetics of pathogens which can integrate its biological, ecological and sociological components. Although various studies have assessed and described the emergence of zoonoses and vector-borne diseases as the result of social-ecological interactions (*e.g.*, [55], [56]), this approach has been disregarded in population and landscape genetic simulations ([2], [49], but see [57]). Integrating social-ecological interactions in landscape genetics remains a key challenge, especially when considering that vectors of pathogens are localized in areas subject to anthropogenic disturbance across scales [58], [59]. At the interface across disciplines, this methodological framework allows the consideration of different types of knowledge and takes into account the causes of vector spatial genetic structure at multiple levels. Our hope is that our study may provide a testable and applicable modeling framework to a broad community of epidemiologists for formulating scenarios of landscape change and foresee their consequences on vector dynamics and genetic structure, with potential implications for their surveillance and control.

## Supporting Information

Supporting Information S1 Material and methods for genotyping studies.
(PDF)
