# Supplementary material for: Simulating Population Genetics of Pathogen Vectors in Changing Landscapes: Guidelines and Application with Triatoma brasiliensis
Source: PLoS Negl Trop Dis. 2014 Aug 7;8(8):e3068. doi: 10.1371/journal.pntd.0003068 (PMC4125301; doi:10.1371/journal.pntd.0003068)
Supplement: Supporting Information S1 — Material and methods for genotyping studies. (PDF) [file pntd.0003068.s001.pdf]

Supporting Information S1: Material and Methods for genotyping studies

Four available primers for *T. brasiliensis* (Harry *et al.* 2009) were used for genotyping studies (Tb728, Tb830, Tb860, and Tb7180), and three additional markers were used after annealing temperature optimization (Tb2146, 52°C; Tb8102, 56°C; Tb8150, 60 to 50°C temperature range using the touch-down strategy). PCR amplification were performed using Dye labelled primer (Applied Biosystem) and the GoTaq® Flexi DNA Polymerase (Promega) in a final volume of 12µl containing 20-50ng of template DNA, 1.5µl of MgCl2 (25mM), 2.5µl of 5X buffer, 0,5µl of dNTP at 10µM each, 0.5µl of each primer at 10µM and 0.05µl of Taq Polymerase at 5U/µl. PCR were performed with 40 cycles (denaturation at 94°C for 30sec, annealing for 30sec, and extension at 72°C for 30sec). Microsatellite data were collected on an ABI Prism 3100 (Applied Biosystem) and alleles analyzed using GeneMapper® (Applied Biosystem) (see details in Harry et al, 2009). Population comparison was made through F_{st} pairwise estimates (Wright, 1943; Slatkin, 1995, computed using ARLEQUIN 3.5.1.2 (Excoffier et Lischer, 2010) using Bonferroni correction to account for all multiple testing (Hochberg 1988).

Excoffier, L., & Lischer, H. E. (2010). Arlequin suite ver 3.5: a new series of programs to perform population genetics analyses under Linux and Windows. *Molecular ecology resources*, *10*(3), 564-567.

Harry, M., Dupont, L., Quartier, M., Diotaiuti, L., Walter, A., Romana, C. (2009). New perspectives for population genetics of Chagas' disease vectors in the Northeastern Brazil: isolation of polymorphic microsatellite markers in *Triatoma brasiliensis*. *Infection Genetic and Evolution 9,* 633-637.

Hochberg ,Y. (1988). A sharper Bonferroni procedure for multiple tests of significance. *Biometrika* 75, 800–802.

Slatkin, M. (1995). A measure of population subdivision based on microsatellite allele frequencies. *Genetics* 139, 457-462.

Wright, S. (1943). Isolation by distance. *Genetics*, 28, 114-138.
